# Estimating Herd Immunity to Amphibian Chytridiomycosis in Madagascar Based on the Defensive Function of Amphibian Skin Bacteria

**DOI:** 10.3389/fmicb.2017.01751

**Published:** 2017-09-13

**Authors:** Molly C. Bletz, Jillian Myers, Douglas C. Woodhams, Falitiana C. E. Rabemananjara, Angela Rakotonirina, Che Weldon, Devin Edmonds, Miguel Vences, Reid N. Harris

**Affiliations:** ^1^Zoologisches Institut, Technische Universität Braunschweig Braunschweig, Germany; ^2^Department of Biology, James Madison University Harrisonburg, VA, United States; ^3^Ecology and Evolutionary Biology, University of Michigan Ann Arbor, MI, United States; ^4^Department of Biology, University of Massachusetts Boston Boston, MA, United States; ^5^Department of Animal Biology, University of Antananarivo Antananarivo, Madagascar; ^6^Laboratoire National de Diagnostic Vétérinaire Antananarivo, Madagascar; ^7^Unit for Environmental Sciences and Management, North-West University Potchefstroom, South Africa; ^8^Illinois Natural History Survey University of Illinois at Urbana-Champaign Champaign, IL, United States

**Keywords:** anti-Bd bacteria, chytridiomycosis, amphibians, skin bacteria, *Batrachochytrium dendrobatidis*

## Abstract

For decades, Amphibians have been globally threatened by the still expanding infectious disease, chytridiomycosis. Madagascar is an amphibian biodiversity hotspot where *Batrachochytrium dendrobatidis* (*Bd*) has only recently been detected. While no *Bd*-associated population declines have been reported, the risk of declines is high when invasive virulent lineages become involved. Cutaneous bacteria contribute to host innate immunity by providing defense against pathogens for numerous animals, including amphibians. Little is known, however, about the cutaneous bacterial residents of Malagasy amphibians and the functional capacity they have against *Bd*. We cultured 3179 skin bacterial isolates from over 90 frog species across Madagascar, identified them via Sanger sequencing of approximately 700 bp of the 16S rRNA gene, and characterized their functional capacity against *Bd*. A subset of isolates was also tested against multiple *Bd* genotypes. In addition, we applied the concept of herd immunity to estimate *Bd*-associated risk for amphibian communities across Madagascar based on bacterial antifungal activity. We found that multiple bacterial isolates (39% of all isolates) cultured from the skin of Malagasy frogs were able to inhibit *Bd*. Mean inhibition was weakly correlated with bacterial phylogeny, and certain taxonomic groups appear to have a high proportion of inhibitory isolates, such as the Enterobacteriaceae, Pseudomonadaceae, and Xanthamonadaceae (84, 80, and 75% respectively). Functional capacity of bacteria against *Bd* varied among *Bd* genotypes; however, there were some bacteria that showed broad spectrum inhibition against all tested *Bd* genotypes, suggesting that these bacteria would be good candidates for probiotic therapies. We estimated *Bd*-associated risk for sampled amphibian communities based on the concept of herd immunity. Multiple amphibian communities, including those in the amphibian diversity hotspots, Andasibe and Ranomafana, were estimated to be below the 80% herd immunity threshold, suggesting they may be at higher risk to chytridiomycosis if a lethal *Bd* genotype emerges in Madagascar. While this predictive approach rests on multiple assumptions, and incorporates only one component of hosts' defense against *Bd*, their culturable cutaneous bacterial defense, it can serve as a foundation for continued research on *Bd*-associated risk for the endemic frogs of Madagascar.

## Introduction

Host-associated symbiotic bacterial communities mediate protection against pathogens in multiple hosts, including plants (Haas and Défago, 2005), corals (Krediet et al., 2013), insects (Cafaro et al., 2011), bats (Hoyt et al., 2015), humans (Sanchez et al., 2016), and amphibians (Bletz et al., 2013; Walke and Belden, 2016). Next generation sequencing technologies have rapidly advanced our understanding of community composition and structure of host microbiotas; however, understanding the function of these communities requires alternative technologies and can be complicated. Culture-based studies can be of great value for determining microbial function. Understanding the functional capacity of culture isolates may help identify phylogenetic patterns of function and thus help to further elucidate how community composition is linked to function.

Bacteria can provide protection against the cutaneous chytrid fungus, *Batrachochytrium dendrobatidis* (*Bd*), which can cause the lethal disease, chytridiomycosis (Berger et al., 1998; Stuart et al., 2004; Lips et al., 2006; Cheng et al., 2011). Resident cutaneous microbes work together with the host's innate immune system to provide a first line of defense against invading pathogens, such as *Bd* (Becker and Harris, 2010). Bacterial symbionts isolated from amphibian skin can inhibit *Bd* growth through the production of anti-fungal compounds (Harris et al., 2006; Brucker et al., 2008a,b; Flechas et al., 2012; Woodhams et al., 2015); however, inhibitory strength of bacterial metabolites can differ among *Bd* genotypes (Antwis et al., 2015). Furthermore, the addition of particular bacterial species, such as *Janthinobacterium lividum*, to the skin of amphibians can increase host survival by reducing the burden of chytridiomycosis (Harris et al., 2009a,b; Vredenburg et al., 2011; Kueneman et al., 2016).

In a study of amphibians from the western USA, population persistence through the emergence of *Bd* has been linked to the proportion of amphibians with *Bd-*inhibitory bacteria residing on their skin (Lam et al., 2010). Lam et al. (2010) propose that a mechanism analogous to herd immunity may, in part, explain variation in population persistence when *Bd* emerges. This concept states that when a given percentage of the population is immunized or protected against a communicable disease, the disease will die out and the population will persist. This critical threshold is a function of an intrinsic property of the pathogen—its reproductive rate (R0). For several amphibian populations and communities, a herd immunity threshold of 80% appears to be a consistent cut-off, below which populations crash when the pathogen emerges, and above which populations persist in coexistence with *Bd* (Woodhams et al., 2007; Lam et al., 2010; Figure S1). Interestingly, for many human diseases, the herd immunity threshold percentage is also around 80% (Anderson and May, 1985; Fine, 1993; Gonçalves, 2008; Fine et al., 2011). From this, a hypothetical model can be derived for further testing: if 80% of amphibian individuals maintain at least one strongly *Bd*-inhibitory bacterium on their skin, the population may persist and coexist with *Bd*.

Madagascar is a hotspot for biodiversity conservation, home to more than 400 frog species, most of which are found nowhere else in the world (Vieites et al., 2009; Perl et al., 2014). Ecological niche modeling suggests that the eastern rainforest of Madagascar is highly suitable for *Bd* (Lötters et al., 2011) and it has a higher amphibian species richness compared to the more arid west (Brown et al., 2016). Until recently, Madagascar was considered naïve to *Bd*, but this pathogen has recently been detected in samples from multiple locations across Madagascar (Vredenburg et al., 2012; Weldon et al., 2013; Bletz et al., 2015). The lineage of *Bd* that is present in Madagascar has not yet been characterized, nor is its virulence known. The potential risk of *Bd*-associated declines is presumed high; however, there is essentially nothing known about the resident bacteria on Madagascar frogs and the role they may have in the hosts' defense against *Bd*. Probiotic therapies have been proposed as a possible disease mitigation strategy for combating chytridiomycosis (Bletz et al., 2013; Walke and Belden, 2016; Woodhams et al., 2016); therefore, culturing and characterizing the function of microbes from amphibian skin works toward a possible mitigation strategy against *Bd*.

We collected samples from over 500 Malagasy frogs from 14 locations, and cultured, sequenced, and characterized the *Bd*-inhibiting functional capacity of over 3,000 bacterial isolates in order to address the following questions: (1) Are bacteria residing on Madagascar frogs able to inhibit *Bd*?, (2) What is the phylogenetic distribution of cultured isolates and does *Bd*-inhibitory function correlate with bacterial phylogeny?, and (3) How does functional capacity of bacteria against *Bd* vary among different *Bd* genotypes? In addition, we use these functional data to estimate *Bd*-associated risk of amphibian communities based on their bacterial defense following the model proposed in Lam et al. (2010). Thus, we ask the questions, (4) Are certain amphibian communities in Madagascar likely to be at risk of developing chytridiomycosis based on their bacterial defense? and (5) Are particular host genera likely to be at risk of developing chytridiomycosis based on their bacterial defense? While bacterial defense makes up only one component of a host's defense against *Bd*, this approach can provide a step toward understanding *Bd*-associated risk for the endemic frogs of Madagascar.

## Methods

### Field sampling

**Figure 1:**
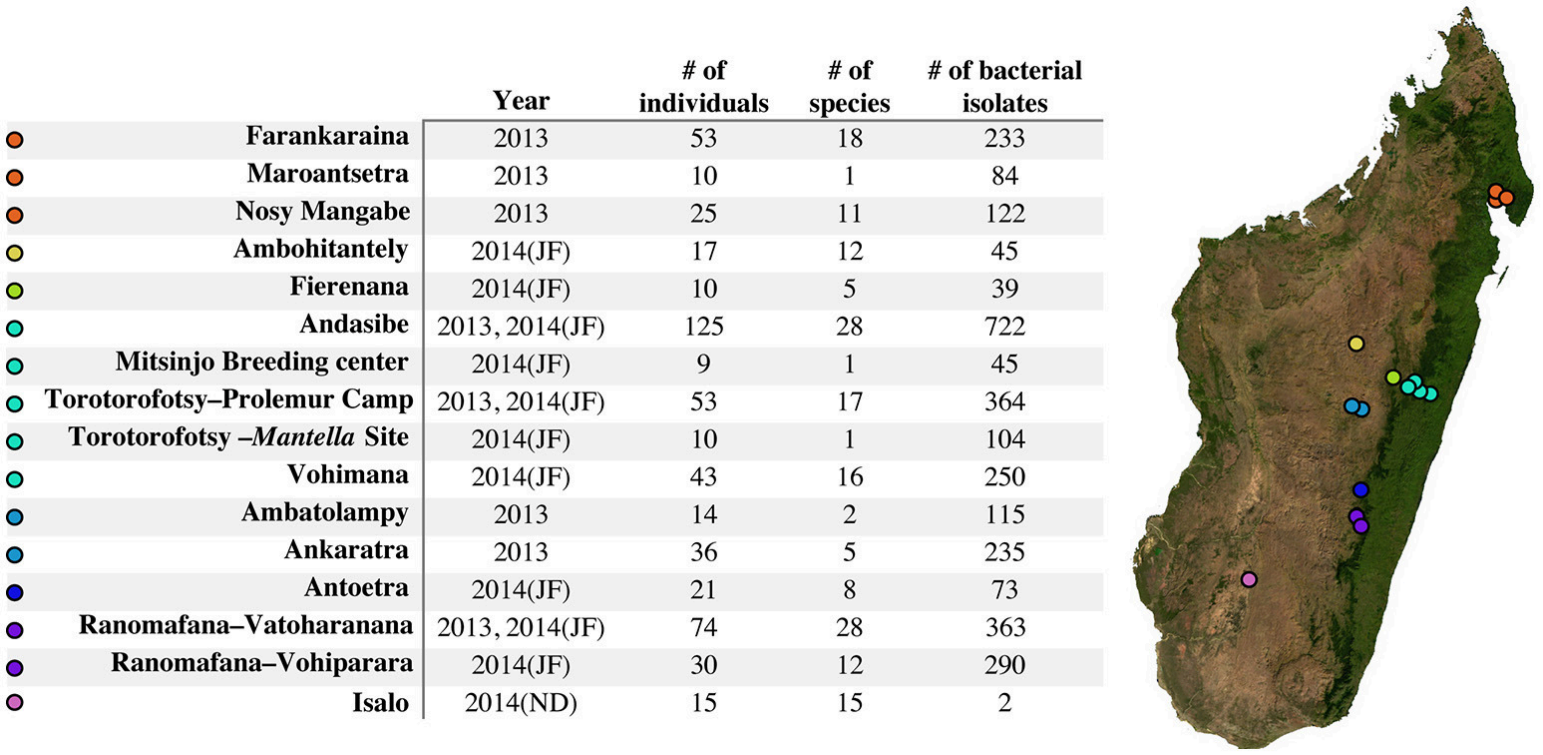
Sampling locations and sample sizes across Madagascar throughout the sampling period. Mitsinjo Breeding center is located in Andasibe (no additional point has been added for this location on the map). Parenthetical “JF” indicates sampling occurred in January–February 2014, and “ND” indicates sampling occurring in November–December 2014. The base map was obtained from www.worldofmaps.net. No permission is required from the copyright holders for the reproduction of this image. Points on the map were generated using Google Earth Pro and afterwards edited on Adobe Illustrator CS6 (Adobe, 2012).

Field sampling took place during three field visits: 14 August–12 September 2013, 4 January–9 February 2014, and 5 November–15 December 2014. In total, 540 culturable skin microbe samples were collected from 14 localities and 93 different host species (Figure 1).

Amphibians were captured during day and night surveys with clean nitrile gloves and were placed in sterile Whirl-Pak® bags (Nasco, Fort Atkinson, WI, USA). For skin microbe sampling, individuals were removed from the bag with a clean pair of nitrile gloves and were rinsed with 50 ml of filter- or UV-sterilized water. After rinsing, individuals were swabbed with 10 strokes on the ventral abdomen, 5 strokes on each ventral thigh, and 5 strokes on each foot using sterile rayon swabs (MW113, Medical Wire Equipment & Co. Ltd., Corsham, UK). Swabs were stored in microcentrifuge tubes containing 100–200 ul of Tryptic-Soy-Yeast-Extract + 20% Glycerol (TSYE+G) and were transported on ice (~4–10°C) until transfer to a −20°C freezer. Frogs were immediately released at the location of capture after sampling. This study was approved by the Institutional Animal Care and Use Committee of James Madison University (protocol #A01-15), and necessary research and access permits were obtained from the Malagasy Direction Générale des Forêts (DGF) and Madagascar National Parks for all sampling.

### Bacterial culturing

Samples were thawed, gently vortexed, and 25 μl of the TSYE+G storage solution was plated on 1% tryptone agar. While using only one culture medium may limit the diversity of bacteria cultured, the one used represents a general low nutrient medium that supports a wide variety of microorganisms, and was used, in part, because it is also a standard one used for culturing *Bd*. Plates were incubated at 21°C for 2 weeks, and were checked every 3 days for morphologically distinct bacterial colonies. For each sample, each morphologically distinct colony was isolated into pure culture, and subsequently was cryopreserved in TSYE+G solution for later *Bd*-growth inhibition testing and 16S rRNA sequencing.

### *Bd*-growth inhibition assays

A *Bd* isolate from the Global Pandemic Lineage (GPL), JEL 423, was used to characterize function of all bacterial isolates. In addition, a subset of 77 isolates (all from Isalo, Madagascar) were tested against a panel of *Bd* isolates, including four GPL isolates from different regions of the world (USA, Panama, Africa, and Australia), as well as one isolate endemic to Brazil, one isolate endemic to Switzerland, and one isolate endemic to Korea (Table 1). Bacterial cell-free supernatant (CFS) obtained from a single liquid culture of each isolate was used for testing against all *Bd* genotypes. Each isolate was tested for its functional capacity against *Bd* using the 96-well assay method described in Bell et al. (2013) and Becker et al. (2015). Briefly, *Bd* zoospores were collected by flooding 3–5 day-old plate cultures with 1% tryptone, allowing zoospores to be released from mature sporangia into the tryptone media. *Bd* zoospores (2 × 10^6^) were grown in the presence of the CFS of each bacterial isolate in triplicate. Bacterial CFS was obtained by filtering a liquid culture grown in co-culture with *Bd* for 3 days on a shaker (250 rpm), through a 0.22 um filter. The following controls were included with each assay in triplicate: (1) positive control–1% tryptone + *Bd* zoospores; (2) nutrient-depleted control–sterile water + *Bd* zoospores; (3) heat-killed control–heat-killed *Bd* zoospores + 1% tryptone; and (4) negative control–1% tryptone only. Assay plates were incubated at 21°C, and growth was measured as optical density (OD) at 492 nm on a spectrophotometer on days 0, 3, and 7.

**Table 1:** Genotypes of *Bd* used for growth-inhibition assays.

***Bd* Genotype**	**Lineage**	**Region**	**Isolated from**	**Isolated by**
JEL 423	GPL	Panama	*Phyllomedusa lemur*	Joyce Longcore
JEL 242	GPL	Africa	*Xenopus*	Joyce Longcore
VMV 813	GPL	Georgia (USA)	*Lithobates catesbeianu*s	Victoria Vasquez
Aus-*L. leseuri*	GPL	Australia	*Litoria lesueri*	Lee Berger
*Bd*-Swiss	CH	Switzerland	*Alytes obstetricans*	Trent Garner
KR *Bombina*-323	Korea	South Korea	*Bombina orientalis*	Arnaud Bataille
Brazil-LFT001/10	Brazil	Brazil	*Hylodes ornatus*	Felipe Toledo

### Bacterial sequencing and identification

DNA was extracted from bacterial isolates using one of three methods: (1) PrepMan Ultra (ThermoFisher Scientific, Waltham, MA, USA), (2) Chelex (Bio-rad, Hercules, CA, USA), or (3) MoBio UltraClean Microbial DNA isolation kit (MoBio, Carlsbad, CA, USA). The PrepMan protocol was as follows: suspend bacterial cells in 100 μl of PrepMan Ultra solution; vortex and incubate for 10 min at 100°C; centrifuge for 3 min at max speed; transfer supernatant to clean tube. The Chelex protocol was as follows: suspend bacterial cells in 100 μl of 5% Chelex solution; vortex and incubate for 20 min at 99°C; centrifuge for 2 min at max speed; transfer supernatant to clean tube. For MoBio extractions, the manufacturer's protocol was followed. Different methods were used to maximize cost efficiency and to extract troublesome bacterial cells.

Polymerase Chain Reactions (PCR) were used with the bacterial primers 27F and 907R to amplify part of the bacterial 16S rRNA gene from the extracted DNA of each bacterial isolate. Amplification was verified using gel electrophoresis, and each isolate was sequenced either using an in-house capillary sequencer (ABI 3130xl) or was sent for sequencing to LGC Genomics in Berlin, Germany. Sequencing produced approximately 500–800 bp for each bacterial isolate. Sequences were cleaned and a preliminary alignment was completed in order to trim to approximately equal lengths (~500–600 bp) in CodonCode Aligner. Trimmed sequences were then aligned with PyNAST in QIIME, and a phylogenetic tree was built using fasttree (Price et al., 2010). The resulting tree was visualized using the Interactive Tree of Life tool (Letunic and Bork, 2007). Taxonomy was assigned to each bacterial isolate with the Ribosomal Database Project Classifier using QIIME (Wang et al., 2007; Caporaso et al., 2010). Sequences were deposited in GenBank (accession numbers GenBank MF523799–MF526895).

### Data analysis

For each tested bacterial CFS, the proportional *Bd* growth was determined by dividing the slope (OD/Time) of *Bd* growth in the presence of a given bacterial CFS by the slope of *Bd* growth in the nutrient-depleted control. Using the nutrient depleted control represents the effect of bacterially-secreted metabolites on *Bd* growth while accounting for the potential effect on growth due to additional nutrients in the culture medium added into the positive control (Bell et al., 2013). This value was subtracted from 1 to obtain a proportional inhibition score for each isolate. Triplicates of each tested bacterial isolate were averaged to obtain a mean inhibition score.

Mantel correlations were used to test the phylogenetic independence of mean inhibition scores. More specifically, distance matrices of the patristic distances between bacterial isolates were compared to distances derived from mean inhibition scores of each isolate. Using the inferred phylogenetic tree, patristic distances between bacterial isolates were calculated with the ape and adephylo packages in R (Paradis et al., 2004; Jombart et al., 2010; R Core Team, 2016). Euclidean distances between mean inhibition scores were calculated in QIIME with the distance_matrix_from_mapping.py script. Mean inhibition was compared across bacterial orders using Kruskal-Wallis tests because the data could not be normalized. Two-way analysis of variance (ANOVA) was used to compare bacterial inhibition of *Bd* across *Bd* genotypes. *Bd* genotype and bacterial isolate ID were the main factors.

To apply the herd immunity model proposed in Lam et al. (2010), the following steps were taken. First, bacterial isolates exhibiting inhibition scores greater than 0.8 (i.e., reduced *Bd* growth by 80%) were considered “inhibitory.” This threshold was chosen because it represents a strong reduction in *Bd* growth, and similar thresholds have been used in other studies (e.g., Becker et al., 2015); note that this value is not related to the threshold in the herd immunity concept associated with the R0, which coincidentally is also 80% (see below). Second, each individual amphibian was classified as protected or not-protected based on the existence of at least one *Bd*-inhibitory isolate cultured from its skin. Next, the proportion of “protected” individuals was determined (1) for each sampled amphibian community (i.e., location) with more than 10 individuals sampled, and (2) for each host genera at two high diversity sites, Andasibe and Ranomafana (that is, the proportion of protected individuals was calculated considering all individuals within a given genus at the particular site). We estimated herd immunity from both the “community” and “host genera” perspective to address it from two scales: (1) a larger scale community framework, and (2) a finer scale examining specific host taxonomic groups within locations. Both frameworks were implemented because they carry different inherent assumptions. For example, variation in protection at the genus or species level could affect community level protection dynamics. For our estimates, we applied the hypothesis of an 80% herd immunity threshold, i.e., we considered a group of amphibians as protected by herd immunity if 80% of the individuals had at least one strongly *Bd*-inhibitory bacterium on their skin. It is important to note that community level investigations make assumptions about potential pathogen transmission dynamics in that they assume contact would be equally likely within or across species (i.e., spatial and contact homogeneity). While contact rates within a species are undoubtedly higher than across species, interspecies or inter-genera contact can be expected to occur frequently given the high spatial overlap and sympatry of amphibians in hyper-diverse locations (i.e. multiple species inhabit small microhabitat areas at relatively high densities). During breeding season when amphibian species congregate at water bodies inter-species contact could be even more probable. In this context, a community level framework can be seen as valid and informative.

## Results

**Figure 2:**
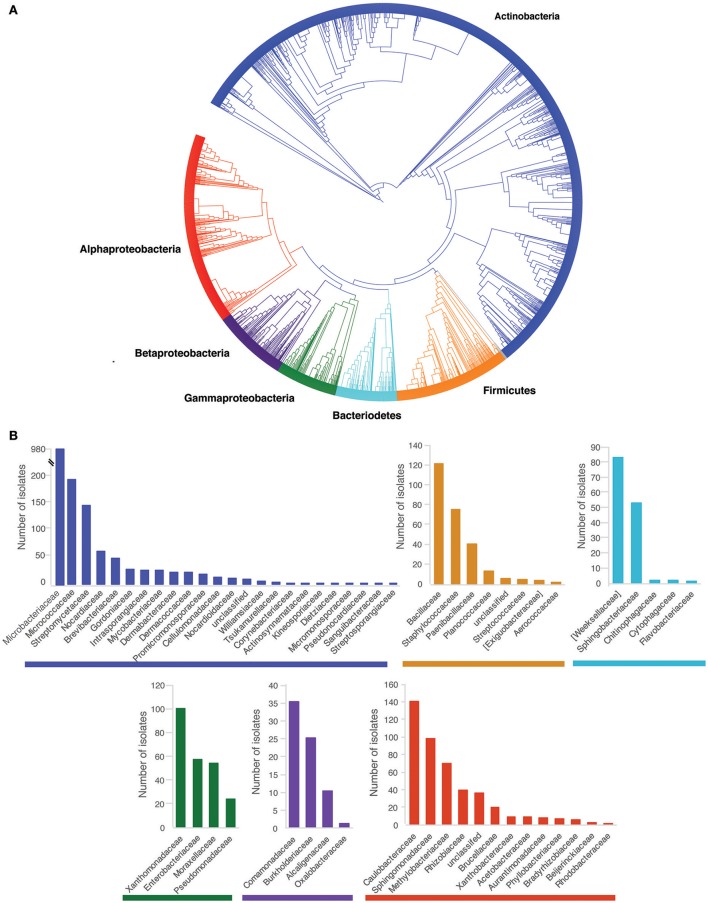
Phylogenetic and taxonomic distribution of cultured isolates. **(A)** Phylogenetic tree of cultured isolates based on DNA sequences of the 16S rRNA gene (~500–600 bp in length). **(B)** Number of cultured isolates within each family of each phylum. Color coding corresponds to bacterial phyla.

From the 540 sampled individuals, 3179 bacterial isolates were cultured and were successfully tested in *Bd*-growth inhibition assays. On average, 7.5 bacterial morphotypes were collected per frog. The cultured isolates were predominantly from the phylum Actinobacteria (57.3%) followed by Proteobacteria (27.1%, [Alpha-59.3%, Gamma-31.2%, Beta-9.4%]), Firmicutes (9.6%), and Bacteriodetes (5.2%) (Figure 2). Inhibitory isolates (reducing *Bd* growth by 80% or more) were identified in all four bacterial phyla represented in the data as well as in all the represented bacterial families (Figure S2).

**Figure 3:**
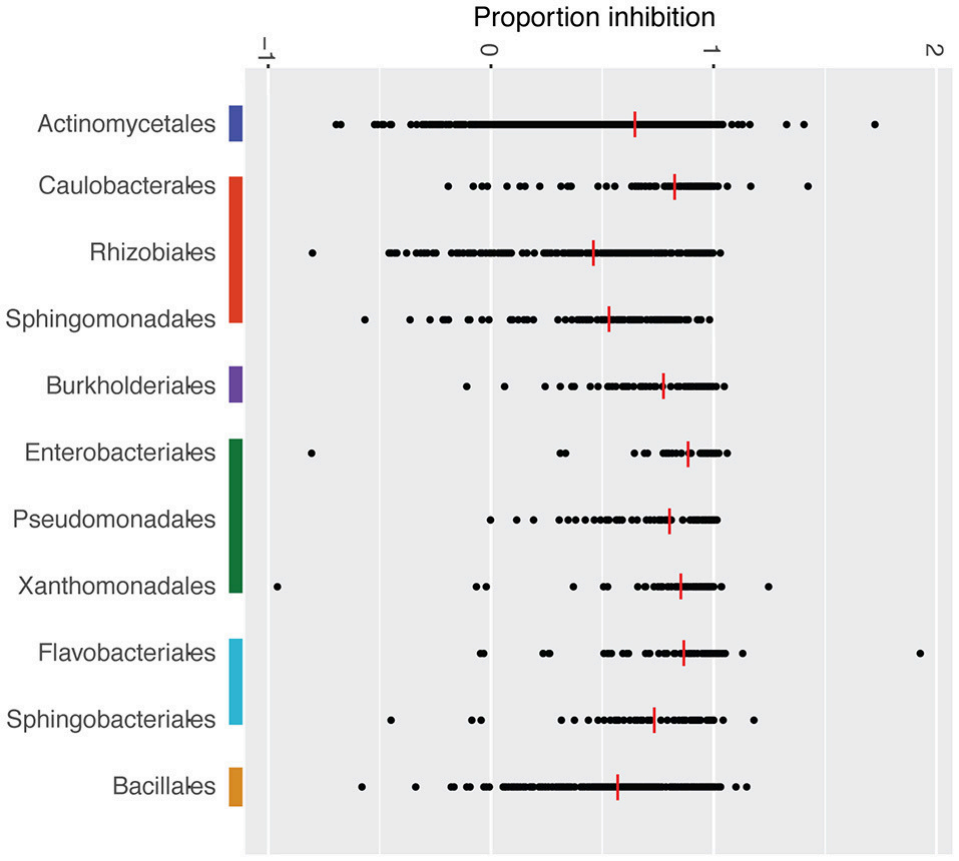
Mean *Bd* inhibition for bacterial isolates within each dominant order. Each black point represents the mean inhibition of a given bacterial isolate, and the red bars represent the mean inhibition score for each order. On the horizontal axis: 1 equals complete inhibition of *Bd* growth; 0 equals no inhibition; values less than 0 indicate facilitation of *Bd* growth. Color bars beside each order name correspond to bacterial phyla in Figure 2.

Mean inhibition was weakly correlated with bacterial phylogeny (Mantel test- *R* = 0.04 *p* = 0.01). Furthermore, mean inhibition varied significantly across bacterial orders (KW chi-squared = 438.2, *df* = 16, *p*-value < 0.001, Figure 3). The orders Caulobacterales, Burkholderiales, Enterobacteriales, Pseudomonadales, Xanthamonadales, and Flavobacteriales consistently exhibited stronger inhibition than the other represented bacterial orders (Figure 3, Table 1). Mean inhibition also varied significantly at the family level (KW chi-squared = 675.2, *df* = 56, *p*-value < 0.001). In some bacterial families, the majority of the isolates were classified as inhibitory, including Caulobacteraceae (102/140), Weeksellaceae (60/83), Enterobacteriaceae (47/57), Pseudomonadaceae (18/24), and Xanthamondaceae (80/100), while other families had only a few inhibitory isolates, including Rhizobiaceae (7/39), Methylobacteriaceae, (4/70) and Staphylococcaceae (6/75) (Figure S2).

Testing of a subset of 77 bacterial isolates cultured from two frog species in Isalo (Mantella expectata: *n* = 57, Scaphiophyrne gottlebei: *n* = 20) showed that inhibition across *Bd* genotypes was not consistent (Table 2). Bacterial inhibition of *Bd* varied significantly among *Bd* genotypes [ANOVA–*F*_(6, 76)_ = 15.46, *p* < 0.001, Table S1).

**Table 2:**
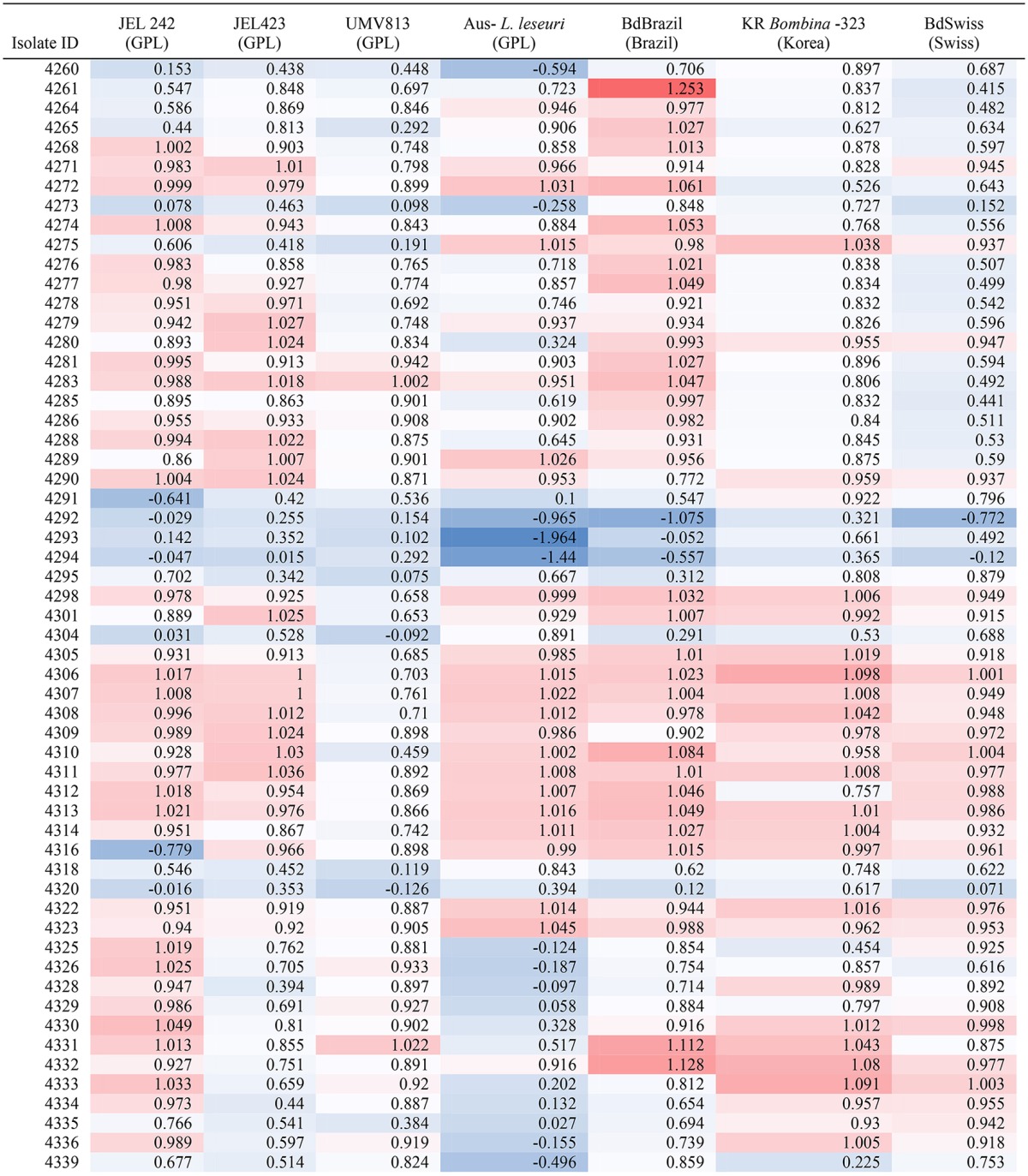
Mean *Bd* inhibition (1 = 100% inhibition of *Bd* growth) across multiple genotypes of *Bd* for the 57 bacterial isolates cultured from *Mantella expectata*. Isolates from *Scaphiophyrne gottlebei* are not shown.

*Gradient of blue-red corresponds to increasing Bd inhibition, with red indicating strong inhibition*.

**Figure 4:**
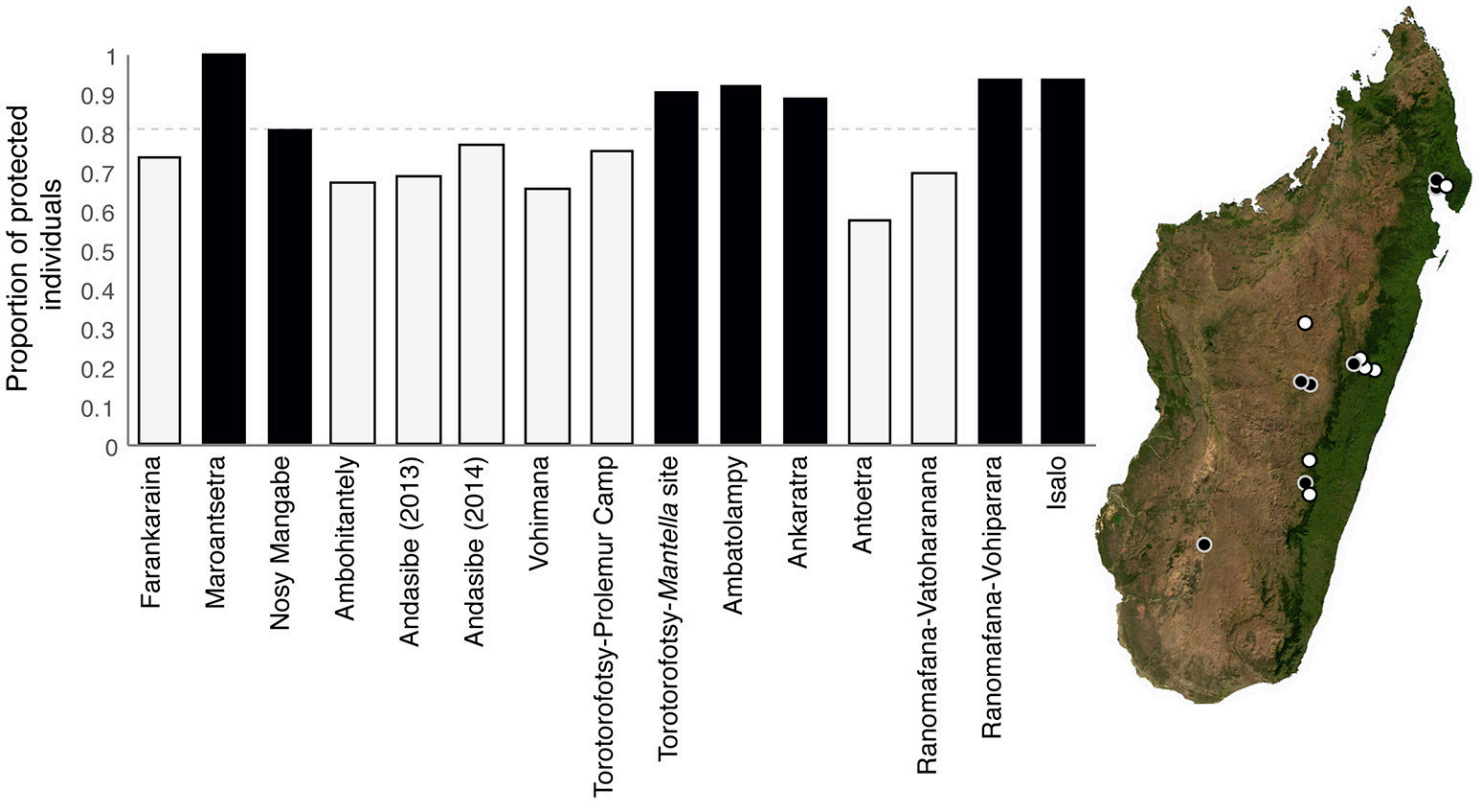
Proportion of “protected” individuals across amphibian communities in Madagascar. Black coloring denotes location that meet or surpass the herd immunity threshold of 80% (i.e., predicted to be protected), and white coloring denotes locations that are below this threshold (i.e. predicted to be at risk). Dotted line represents the herd immunity threshold (80%). Map on the right shows the distribution of “protected” and “unprotected” locations across Madagascar. The base map was obtained from www.worldofmaps.net. No permission is required from the copyright holders for the reproduction of this image. Points on the map were generated using Google Earth Pro and afterwards edited on Adobe Illustrator CS6 (Adobe, 2012).

**Figure 5:**
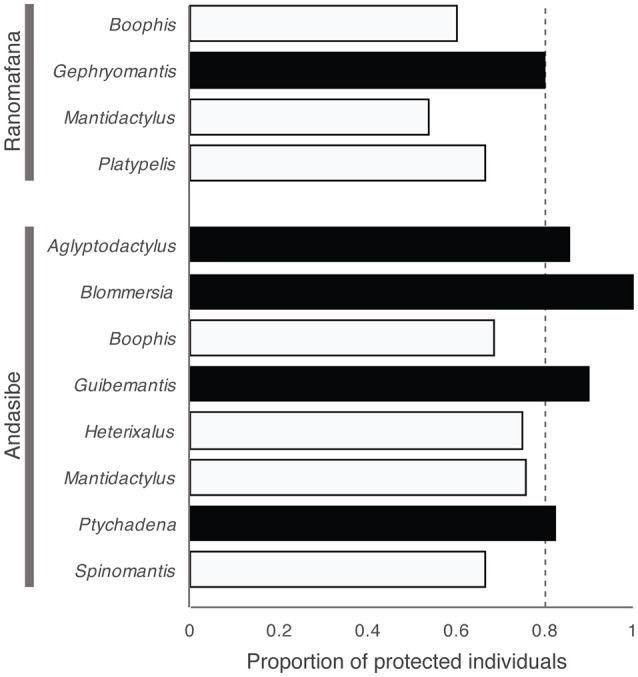
Proportion of “protected” individuals across host genera at two hyperdiverse sites in Madagascar. Black coloring denotes genera that meet or surpass the herd immunity threshold of 80% (i.e., predicted to be protected), and white coloring denotes genera that are below this threshold (i.e., predicted to be at risk). Dotted line represents the herd immunity threshold (80%).

We determined the proportion of protected individuals within each amphibian community (i.e., each sampled locations) and within each host genus at Andasibe and Ranomafana by defining an individual as “protected” if at least one of its cultured isolates was classified as inhibitory. Proportion of protected individuals differed across locations, ranging from 57 to 100%. Locations predicted to be unprotected included Farankaraina, Ambohitantely, Andasibe, Torotorofotsy, Antoetra, and Ranomafana-Vatoharanana, while Nosy Mangabe, Maroantsetra, Ankaratra, Ambatolampy, Ranomafana-Vohiparara, and Isalo were predicted to be protected (Figure 4). Proportion of protected individuals also varied among host genera at both selected sites. In Ranomafana, our predictions suggest that *Boophis, Mantidactylus*, and *Platypelis* fall below the herd immunity threshold, while *Gephyromantis* meets this threshold. In Andasibe, *Boophis, Mantidactylus, Spinomantis*, and *Heterixalus* fell below the herd immunity threshold, while the genera *Aglyptodactylus, Blommersia*, and *Ptychadena* surpassed this threshold (Figure 5).

## Discussion

### Phylogenetic and taxonomic distribution of *Bd*-inhibitory function

*Bd* inhibition by bacterial isolates derived from the skin of Malagasy amphibians was widespread across the bacterial phylogenetic tree, but mean inhibition was weakly correlated with bacterial phylogeny, suggesting that anti-*Bd* function may be at least in part phylogenetically conserved. This finding differs from that of a Panamanian frog skin bacteria study where inhibition was not correlated with bacterial phylogeny (Becker et al., 2015). The correlation herein was rather weak (Mantel R statistic = 0.04, but *p*-value of 0.01); therefore, bacterial phylogeny is likely not the main driver of inhibitory function against *Bd*, which could be associated with the highly flexible genomes of bacteria (Fuhrman, 2009). Bacterial genes can be readily transferred via horizontal gene transfer (Smillie et al., 2011). In fact, different bacterial species have been observed to transfer genes encoding for antifungal compounds (Ravel et al., 2000). Different bacterial taxa are also known to produce the same anti-fungal compounds, and this is even the case for known *Bd*-inhibitory compounds. For example, 2,4 DAPG is produced by both *Pseudomonas* (Pseudomonadaceae) and *Lysobacter* (Xanthamonadaceae), and violacein is produced by multiple taxa spanning across different bacterial genera including *Janthinobacterium, Collimonas, Duganella, Pseudoalteromonas*, and *Microbulbifer* (Brucker et al., 2008b; Choi et al., 2015).

Inhibitory taxa were found within all major bacterial orders and families which mirrors the findings of other studies (Harris et al., 2006; Woodhams et al., 2007; Flechas et al., 2012; Becker et al., 2015). Despite the fact that *Bd* inhibition was documented across nearly all taxonomic groups, certain bacterial groups appear to be composed of mainly inhibitory isolates [e.g., Caulobacteraceae (72%), Weeksellaceae (73%), Enterobacteriaceae (82%), Pseudomonadaceae (80%), and Xanthamondaceae (80%)] which might be responsible for the weak phylogenetic effect found in our overall data set. Many of these groups have been identified to have high proportions of inhibitory isolates in other amphibian studies as well (Becker et al., 2015). Pseudomonads, in particular, are well-documented in other systems including plants, crustaceans, fish, and bats as having pathogen-inhibiting effects (Spanggaard et al., 2001; Ramette et al., 2003, 2011; Balcázar et al., 2007; Kim et al., 2007; Cheng et al., 2016). Additionally, it is important to note that *in vitro* assays do not directly indicate *in vivo* function; other biotic and abiotic factors can influence the functional behavior of bacteria on amphibian skin.

### Estimating *Bd*-associated risk based on bacterial defense

The functional characterization of the resident bacteria's ability to inhibit *Bd* was used to estimate the potential risk or susceptibility to *Bd*-associated declines in the context of the herd-immunity model proposed by Lam et al. (2010). This model is based on results from the *Rana muscosa*/*sierrae* system in the USA and from consideration of the concept of herd immunity in other systems. A population found co-existing with *Bd* had 80% of individuals with at least one *Bd*-inhibitory isolate, and a population below this 80% threshold was declining once *Bd* emerged in the population (Woodhams et al., 2007). In addition, a naïve population that met the 80% threshold did not go extinct while other naïve populations in this region went extinct (Lam et al., 2010). Several additional studies support this model (Figure S1).

While predictions within this framework provide an integrative look at how protection provided by bacteria varies across amphibian taxa and locations in Madagascar, it is important to note the following limitations of the model: (i) only the culturable community is considered, (i) bacterial interactions (antagonistic or synergistic) are not considered, (iii) the bacterial function assessment is based on high density *in vitro* testing (i.e., the hypothesis of antifungal activity does not take the *in vivo* density of a specific bacterium into account), and (iv) predictions are based on one GPL *Bd* isolate only. In general, dominant bacteria associated to the amphibian skin can be cultured with common techniques (Walke et al., 2015). To further support this within our study system, a comparison with data from Illumina-based sequencing of bacterial 16S amplicons from the skin of Malagasy amphibians (Bletz et al., 2017) suggests that a relatively high proportion of the dominant community members are represented among the cultured isolates [75% of top 80 illumina OTUs were present in the cultured isolates (Bletz, personal observation)]. Nevertheless, because additional (uncultured) members of the community might also inhibit *Bd*, our assessment of the number of protected individuals is conservative, and more individuals than estimated may have at least one *Bd*-inhibiting bacterium on their skin.

Our predictions based on the herd immunity model suggest that risk of developing chytridiomycosis varies across the landscape in Madagascar. Amphibian communities at some locations appear protected (above 80%), while others fall below this herd-immunity threshold. Locations predicted to be unprotected included Farankaraina, Ambohitantely, Andasibe, Torotorofotsy, Antoetra, and Ranomafana-Vatoharanana, while Nosy Mangabe, Maroantsetra, Ankaratra, Ambatolampy, Ranomafana-Vohiparara, and Isalo appear to be protected. There is no clear biogeographical or ecological pattern in the protected vs. unprotected categories; both contain localities from low-, mid-, and high-elevations, from eastern humid regions, and from drier regions of the central plateau (Brown et al., 2016). However, the fact that locations like Andasibe, Torotorofotsy, and Ranomafana-Vatoharanana are predicted to be at risk is particularly concerning considering these are all hyperdiverse mid-high elevation rainforest sites also predicted by ecological niche modeling to be highly suitable for *Bd* (Lötters et al., 2011). These sites are ecologically similar to places in Central America where drastic populations declines have occurred (La Marca et al., 2005; Lips et al., 2006). In addition, locations, such as Ambohitantely and Antoetra, are home to critically endangered species (*Anodonthyla vallani* and *Mantella cowani*, respectively) that have restricted distributions. It is important to note that our community predictions are based of variable numbers of species within the sampled locations and protection may vary non-randomly across host species or genera (see below); thus, these results should be taken as a preliminary look and continued research is needed to investigation complex community–infection dynamics that may occur in diverse amphibian assemblages.

Proportions of protected individuals also differed across host genera in Andasibe and Ranomafana, suggesting that Bd-associated risk would not be equal across amphibian hosts. Our predictions suggest that *Boophis, Mantidactylus, Platypelis, Spinomantis*, and *Heterixalus*, may be more at risk, at least at the sampled locations, while the genera *Aglyptodactylus, Blommersia, Guibemantis, Gephyromantis*, and *Ptychadena* are predicted to be protected. Interestingly, all of the potentially protected species, except *Gephyromantis*, are pond-breeding species, whereas the majority of *Boophis* and *Mantidactylus*, as well as all *Spinomantis*, are stream breeders. In general, stream-breeding amphibians are considered more susceptible to chytridiomycosis (Stuart et al., 2004). We hypothesize that genera such as *Aglyptodactylus, Blommersia, Guibemantis*, and *Ptychadena* (e.g., the pond breeders) might be protected against this disease by two mechanisms: (1) due to a high-proportion of individuals possessing *Bd*-inhibiting cutaneous bacteria, and (2) due to their microhabitat preferences, which include periodic stays in or near warm, stagnant water bodies that do not provide suitable conditions for survival of *Bd* (Kriger and Hero, 2007; Forrest and Schlaepfer, 2011). In addition, *Aglyptodactylus* and *Ptychadena*, and partly *Blommersia* and *Gephyromantis*, are ground-dwelling frogs, and perhaps their association with terrestrial habitats increases the abundance of transient and established fungal-inhibiting bacteria on their skin, as soil is known to be a species-rich and functionally diverse environment (Torsvik and Øvreås, 2002).

### Toward probiotics for Malagasy amphibians

**Figure 6:**
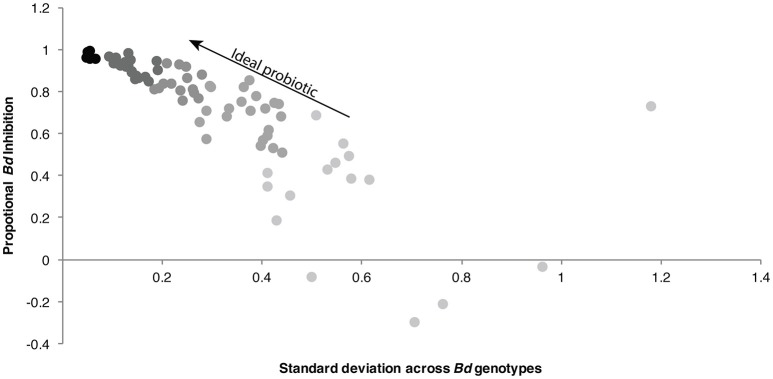
Selecting bacterial isolates with functional consistency across *Bd* genotypes. Scatterplot displays mean inhibition vs. standard deviation of inhibition across *Bd* genotypes. The ideal probiotic candidates will be those with strong inhibitory function and low standard deviation. Points are colored from gray to black to illustrate increasing potential effectiveness as a probiotic.

Probiotic therapies have been proposed as a possible disease mitigation strategy for combating chytridiomycosis (Bletz et al., 2013; Walke and Belden, 2016; Woodhams et al., 2016). To establish such therapies, culturing and characterizing function of microbes from amphibian skin is an important first step. One of the main objectives of this research was to evaluate the functional capacity of bacteria isolated from Madagascar frogs against *Bd*, and to determine whether *Bd*-inhibitory taxa are present. Indeed, inhibitory bacterial taxa were cultured: 39% (1241 isolates) of the cultured isolates inhibited *Bd* by at least 80%, and 26% (829 isolates) inhibited *Bd* by at least 90%. These strongly inhibitory taxa can all serve as potential probiotic candidates for Madagascar's frogs if a lethal *Bd* genotype arrives in Madagascar. *Bd*-inhibition was found herein and in other studies (Antwis et al., 2015) to vary across *Bd* genotypes; that is, not all bacteria could consistently inhibit a panel of *Bd* genotypes, which has important implications for development of probiotic disease mitigation strategies. Ideal probiotics will be bacterial isolates that do demonstrate broad spectrum *Bd*-inhibitory function; that is, they have high inhibition scores (>80%) across *Bd* variants, and have a low standard deviation across replicates (Figure 6). While function across *Bd* genotypes varied for the bacterial isolates tested, there were some bacterial isolates with broad-spectrum *Bd*-inhibitory function, such as a *Chryseobacterium trutae*, a *Elizabethkingia miricola*, a *Pedobacter nutrimenti*, and a *Delftia acidovorans* (Table S2).

These results serve as a basis for continued development of probiotic disease mitigation strategies for the frogs of Madagascar by providing a bank of potential probiotics. In addition, they provide an initial estimate of *Bd*-associated risk across Madagascar, which can facilitate prioritization of locations and host genera that appear to be more at risk. It is important to acknowledge that bacterial defense is only one component of a host's defense against *Bd*; therefore, our predictive approach must be taken as preliminary hypothesis, and as a stimulus for future research on *Bd*-associated risk for the frogs of Madagascar. Continued research on host protection (bacterial and host-produced defenses) against disease is needed to improve our understanding of disease risk across the landscape in Madagascar and help inform integrative conservation management planning. Future research should continue along the probiotic selection steps outlined in Bletz et al. (2013) by working toward identifying which *Bd*-inhibitory taxa can colonize and persist on frog hosts. Bioaugmentation as a mitigation tool requires a deeper understanding of bacterial community assembly and stability in the context of the amphibian host community and their skin secretions (Garner et al., 2016), which will be an important part of future research. Before probiotics can be widely implemented as a long-term management strategy for wild amphibian populations broader aspects including the potential risk probiotics pose to ecosystems and public health must been assessed (Woodhams et al., 2016). However, provided that candidate bacteria meet the necessary criteria, bioaugmentation could be far more cost-effective, ethical and less controversial than the current alternative treatment, namely chemicals (Garner et al., 2016). Continued probiotic research will bring us one step closer to an integrative probiotic approach for mitigating possible *Bd*-associated declines in Madagascar.

## Author contributions

MB and RH designed project with significant input from FR, CW, DE, and MV. MB, FR, AR, CW, DE and RH conducted sampling. MB, JM, and AR performed laboratory work. MB completed data analysis and wrote the paper. All authors contributed to revision of the manuscript and have approved the final manuscript.

### Conflict of interest statement

The authors declare that the research was conducted in the absence of any commercial or financial relationships that could be construed as a potential conflict of interest.
