# Supplementary material for: Estimating Herd Immunity to Amphibian Chytridiomycosis in Madagascar Based on the Defensive Function of Amphibian Skin Bacteria
Source: Front Microbiol. 2017 Sep 13;8:1751. doi: 10.3389/fmicb.2017.01751 (PMC5604057; doi:10.3389/fmicb.2017.01751)
Supplement: Supplementary file 1 [file Table1.pdf]

Supplementary Material

Estimating herd immunity to amphibian chytridiomycosis in Madagascar based on the defensive function of amphibian skin bacteria

Molly C Bletz[1,2], Jillian Myers[3], Douglas C Woodhams[4], Falitiana CE Rabemananjara[5], Angela Rakotonirina[6], Che Weldon[7], Devin Edmonds[8], Miguel Vences[1], Reid N Harris[2]

Supplementary Table 1. Results of pair-wise Wilcoxon test comparing *Bd* inhibition among bacterial orders for cultured isolates from the skin of frogs from Madagascar.

	Actinomycetales	Bacillales	Burkholderiales	Caulobacterales	Enterobacteriales	Flavobacteriales	Pseudomonadales	Rhizobiales	Sphingobacteriales	Sphingomonadales
Bacillales	<0.001	-	-	-	-	-	-	-	-	-
Burkholderiales	<0.001	<0.001	-	-	-	-	-	-	-	-
Caulobacterales	<0.001	<0.001	0.178	-	-	-	-	-	-	-
Enterobacteriales	<0.001	<0.001	<0.001	<0.001	-	-	-	-	-	-
Flavobacteriales	<0.001	<0.001	<0.001	0.022	0.588	-	-	-	-	-
Pseudomonadales	<0.001	<0.001	0.242	0.957	0.012	0.081	-	-	-	-
Rhizobiales	<0.001	0.021	<0.001	<0.001	<0.001	<0.001	<0.001	-	-	-
Sphingobacteriales	0.017	<0.001	0.574	0.034	<0.001	0.002	0.142	<0.001	-	-
Sphingomonadales	<0.001	0.772	<0.001	<0.001	<0.001	<0.001	<0.001	0.148	<0.001	-
Xanthomonadales	<0.001	<0.001	0.065	0.534	0.006	0.111	0.684	<0.001	0.004	<0.001
