# Supplementary material for: Estimating Herd Immunity to Amphibian Chytridiomycosis in Madagascar Based on the Defensive Function of Amphibian Skin Bacteria
Source: Front Microbiol. 2017 Sep 13;8:1751. doi: 10.3389/fmicb.2017.01751 (PMC5604057; doi:10.3389/fmicb.2017.01751)
Supplement: Supplementary file 2 [file Table2.pdf]

Supplementary Material

Estimating herd immunity to amphibian chytridiomycosis in Madagascar based on the defensive function of amphibian skin bacteria

Molly C Bletz[1,2], Jillian Myers[3], Douglas C Woodhams[4], Falitiana CE Rabemananjara[5], Angela Rakotonirina[6], Che Weldon[7], Devin Edmonds[8], Miguel Vences[1], Reid N Harris[2]

Supplementary Table 2: Bacterial taxonomic information for isolates characterized as 'ideal probiotics' based on high *Bd*-inhibitory function and low standard deviation of inhibition among *Bd* genotypes (Supplementary Figure 2).

Isolate ID	Bacterial species	Average inhibition score (+ SD)	Accession number of closest NCBI match	percen t match
4309	*Chryseobacterium trutae* (Flavobacteriia)	0.946+0.052	NR_108531.1	100
4311	*Elizabetkingia miracola* (Flavobacteriia)	0.987+0.051	NR_036862.1	99
4313	*Elizabetkingia miracola* (Flavobacteriia)	0.989+0.057	NR_036862.1	99
4323	*Pedobacter nutrimenti* (Sphingobacteria)	0.959+0.049	NR_133811.1	98
1622	*Delftia acidovorans* (Betaproteobacteria)	0.954+0.069	NR_113708.1	100
