# Supplementary material for: Estimating Herd Immunity to Amphibian Chytridiomycosis in Madagascar Based on the Defensive Function of Amphibian Skin Bacteria
Source: Front Microbiol. 2017 Sep 13;8:1751. doi: 10.3389/fmicb.2017.01751 (PMC5604057; doi:10.3389/fmicb.2017.01751)
Supplement: Supplementary file 3 [file Image1.pdf]

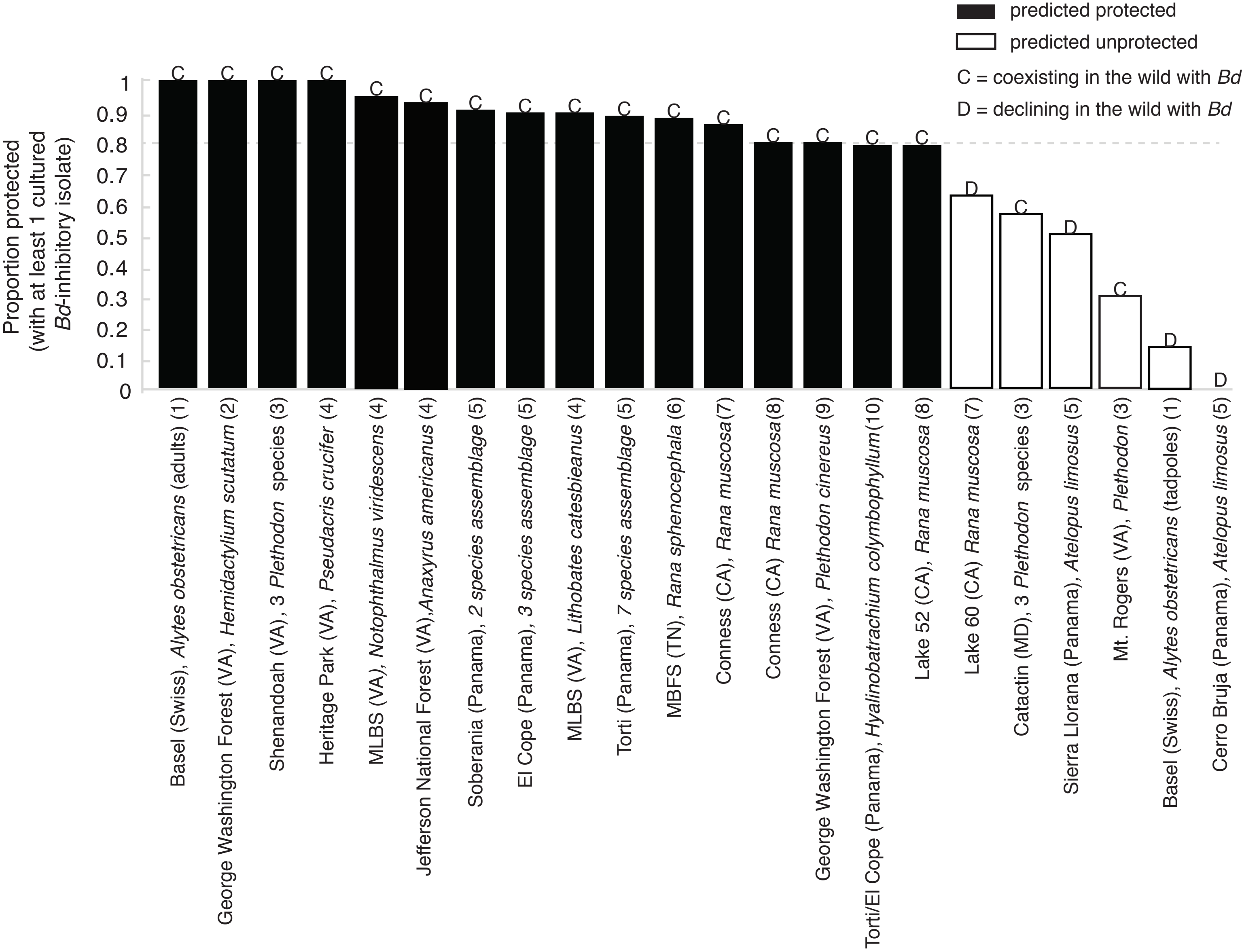

Supplementary Figure 1: Herd immunity predictions and population status for published studies on cultured amphibian skin bacteria. Shading (white/black) of bars represents the prediction of protection based on the herd immunity threshold of 80% with black indicating predicted to be protected and white indicating predicted to be unprotected. Letters (C/D) represent the population status outcome in the presence of *Bd* with "C" representing co-existing and "D" presenting "declining". Parenthetical numbers indicate the published studies: 1 - Woodhams et al. 2014 & Davis et al. 2017; 2 - Lauer et al. 2008; 3 - Muletz-Wolz et al. 2017; 4 - Walke et al. 2015; 5- Becker et al. 2015; 6 - Holden et al. 2015; 7- Woodhams et al. 2007; 8 - Lam et al. 2010; 9 - Lauer et al. 2007; 10 - Walke et al. 2011. Mountain Lake Biological Station is abbreviated with MLBS. Meeman Biological Field Station is abbreviated as MBFS.
