# Supplementary material for: Estimating Herd Immunity to Amphibian Chytridiomycosis in Madagascar Based on the Defensive Function of Amphibian Skin Bacteria
Source: Front Microbiol. 2017 Sep 13;8:1751. doi: 10.3389/fmicb.2017.01751 (PMC5604057; doi:10.3389/fmicb.2017.01751)
Supplement: Supplementary file 4 [file Image2.pdf]

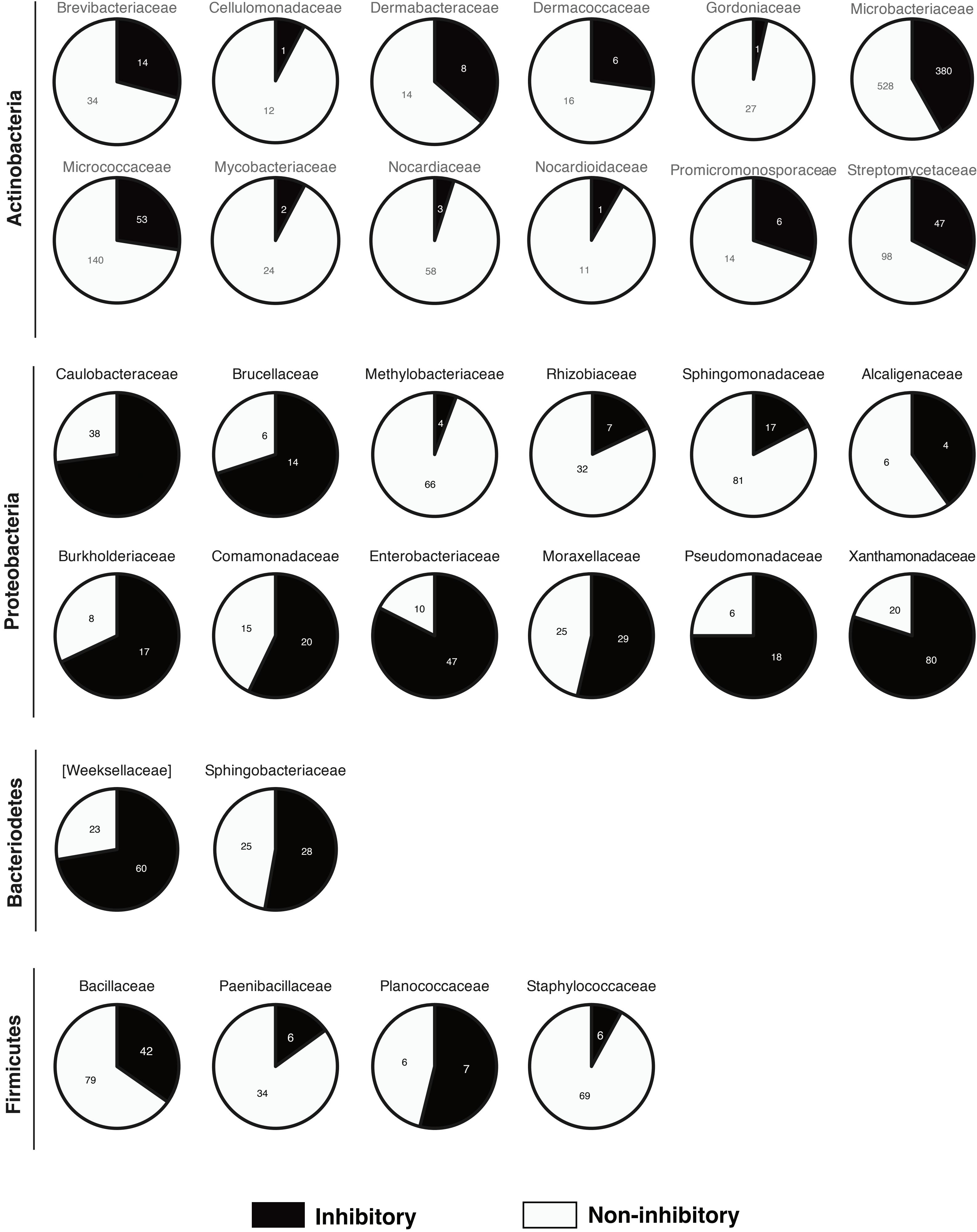

Figure S2. Proportion of inhibitory isolates across dominant bacterial families. Pie charts are given for each bacterial family. Families with less than 10 isolates are omitted. Numbers within each chart individual the number of isolates in the respective inhibition category. Inhibitory is defined as greater than 80% inhibition of *Bd* growth.
